# Application of acoustic agglomeration to enhance air filtration efficiency in air-conditioning and mechanical ventilation (ACMV) systems

**DOI:** 10.1371/journal.pone.0178851

**Published:** 2017-06-08

**Authors:** Bing Feng Ng, Jin Wen Xiong, Man Pun Wan

**Affiliations:** School of Mechanical and Aerospace Engineering, Nanyang Technological University, Singapore, Singapore; Universita degli Studi della Tuscia, ITALY

## Abstract

The recent episodes of haze in Southeast Asia have caused some of the worst regional atmospheric pollution ever recorded in history. In order to control the levels of airborne fine particulate matters (PM) indoors, filtration systems providing high PM capturing efficiency are often sought, which inadvertently also results in high airflow resistance (or pressure drop) that increases the energy consumption for air distribution. A pre-conditioning mechanism promoting the formation of particle clusters to enhance PM capturing efficiency without adding flow resistance in the air distribution ductwork could provide an energy-efficient solution. This pre-conditioning mechanism can be fulfilled by acoustic agglomeration, which is a phenomenon that promotes the coagulation of suspended particles by acoustic waves propagating in the fluid medium. This paper discusses the basic mechanisms of acoustic agglomeration along with influencing factors that could affect the agglomeration efficiency. The feasibility to apply acoustic agglomeration to improve filtration in air-conditioning and mechanical ventilation (ACMV) systems is investigated experimentally in a small-scale wind tunnel. Experimental results indicate that this novel application of acoustic pre-conditioning improves the PM_2.5_ filtration efficiency of the test filters by up to 10% without introducing additional pressure drop. The fan energy savings from not having to switch to a high capturing efficiency filter largely outstrip the additional energy consumed by the acoustics system. This, as a whole, demonstrates potential energy savings from the combined acoustic-enhanced filtration system without compromising on PM capturing efficiency.

## 1. Introduction

The annual episodes of haze in the Southeast Asia region resulting from forest fires engulf the air with fine particulate matters (PM), with conditions worsening in recent years due to the dry weather conditions brought about by the El Niño southern oscillation and southwest monsoon [1]. The thick atmospheric particle loading obstructs visibility and brings about detrimental effects to health [2–5]. During these periods, people spend considerably more time indoors and widely rely on mechanical filtration systems in buildings to maintain a healthy environmental quality, which inadvertently increases energy demand [6]. It is estimated that buildings account for 20% to 40% of the total energy consumption in developed countries and is projected to grow by 56% in the next 30 years [7]. In buildings, the operation of air-conditioning and mechanical ventilation (ACMV) systems accounts for nearly half of the total building electricity consumption [8,9] and this can be as high as 70% for buildings in the tropics [10] or even higher during episodes of haze [6].

In a typical ACMV system, around 15% to 30% of the system energy consumption is devoted to air distribution and most of it is consumed by fans to overcome losses (pressure drop) in the distribution ducts and across filters [11,12]. Meanwhile, the air filters are necessary to reduce human exposure to indoor air pollutants such as suspended PM and bioaerosols [13–15]. In order to reduce energy consumption, yet maintaining the filtration capabilities of ACMV systems, a possible solution is to pre-condition the PM such that they shift to sizes that are more effectively captured by the filters. The strategy proposed is the use of acoustic agglomeration to manipulate the motion of airborne particles to promote coagulation prior to filtration.

**Fig 1:**
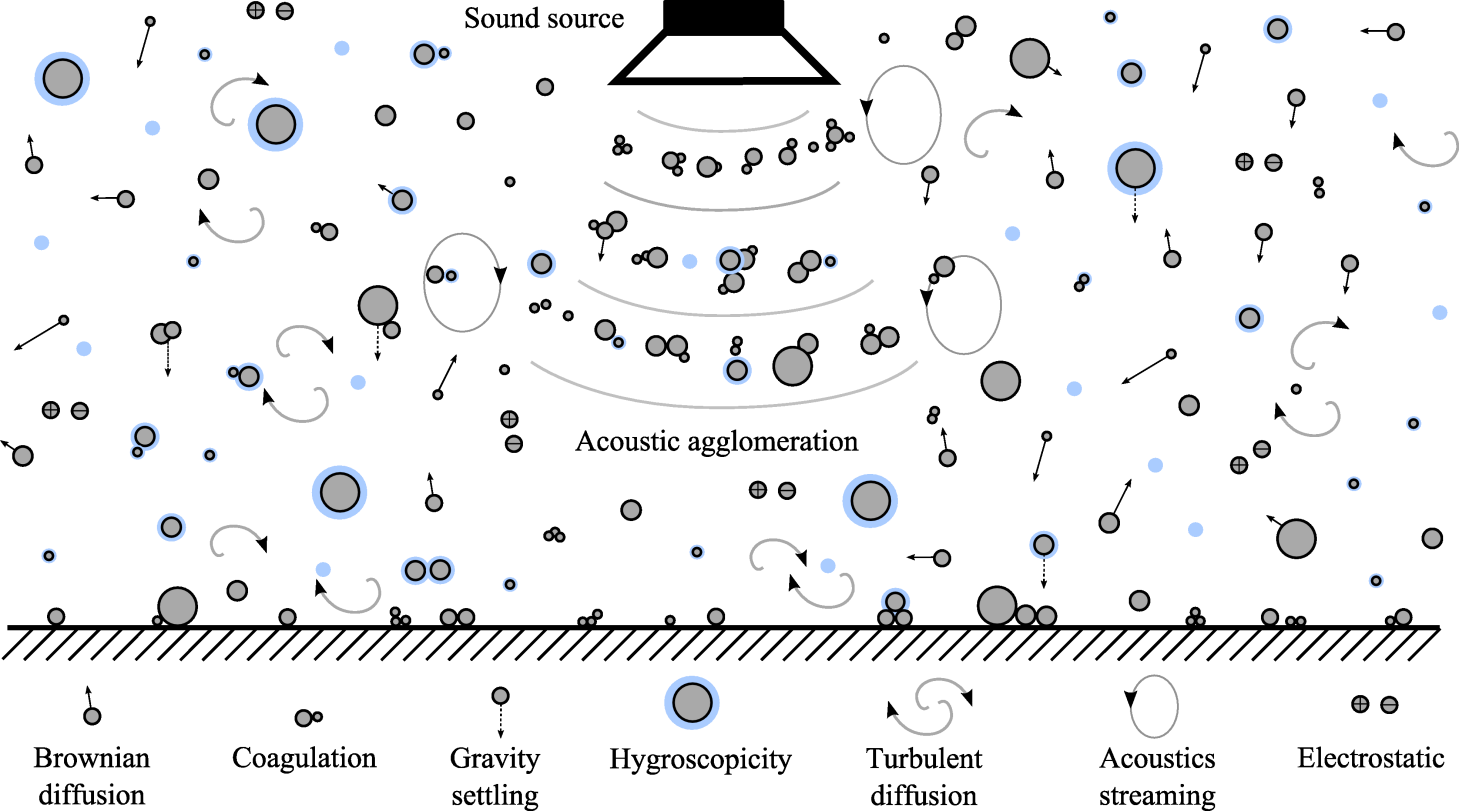
Airborne particle transport mechanisms. Particle transport mechanisms that include Brownian diffusion, coagulation, gravity settling, hygroscopicity, turbulent diffusion, acoustic streaming and electrostatic. Acoustics agglomeration is the forced mechanism induced by acoustic waves.

Acoustic agglomeration is the process in which acoustic waves are used to manipulate the motion of airborne particles [16–20] and in the process, promote collisions that lead to the formation of agglomerates (clustering of particles). The newly-formed clusters continue to agglomerate with others and results in a cascading growth of particles. Among the forces acting on airborne fine particles, as summarised in Table 1, the magnitude of pressure gradient forces resulting from acoustics is significantly greater than inertia forces, thus enabling fine particles to oscillate with the acoustic waves to promote agglomeration. Additionally, among the different transport mechanisms for suspended particles, illustrated in Fig 1, acoustic agglomeration is a forced mechanism where the average increase in particle radius after 1 second and 5 seconds of exposure to acoustic waves could be as high as one order and two orders of magnitude, respectively [21].

**Table 1:** Forces acting on fine particles (PM_2.5_) in an acoustics field.

Forces	Magnitude (N)
Viscous forces [22–24]	10^−3^ to 10^−5^
Pressure gradient forces (acoustics) [24–26]	10^−11^ to 10^−12^
Inertia forces [23,24]	10^−15^ to 10^−18^

The acoustic agglomeration technology has been adopted for treatment of high concentration particles in liquid and gaseous mediums in industries such as chemical processing, oil & gas, food production and control of environmental pollution [27,28]. The focus has been on fine particles that makes up a significant portion of particle emissions that is difficult to be removed using other separation technologies. For instance, cyclone separators used in the control of industrial burning emissions have efficiencies less than 40% for particles below 5 μm [29–32]. By introducing acoustic pre-conditioning, fine particles form clusters that are large enough to be removed effectively through cascades of cyclone stages. Apart from industrial emissions control, there is also the potential for applications in ACMV systems for PM and bioaerosols removal where typical filter efficiency is low for particles in the size range of 0.01 μm to 2 μm [33,34]. The hypothesis is that fine PM and bioaerosols could be pre-conditioned by means of acoustic agglomeration to form larger clusters that can be removed more easily by filtration through the mechanisms of interception and inertial impaction in ACMV systems. This can be achieved without the need to switch to higher efficiency filters that cause higher airflow resistance. In a recent study by Zhou et al. [35], the acoustic agglomeration (1.4 kHz and 148 dB) technique was shown to improve the mass removal efficiency of in-house developed bag filters by up to 99%.

In this paper, the mechanics of acoustic agglomeration encompassing the orthokinetic, hydrodynamic, streaming and other secondary effects are discussed. Following which, the different influencing parameters including particle characteristics and acoustic parameters based on studies reported in the literature are summarised. This provides the fundamental understanding of the acoustic agglomeration phenomenon for subsequent application of the technology in ACMV systems. Feasibility of application in ACMV system is demonstrated through experiments conducted in a small-scale wind tunnel, along with discussions on the findings and challenges in adopting the concept as a pre-conditioner for particle filtration in ACMV settings.

## 2. Mechanics of acoustic agglomeration

The phenomenon of acoustic agglomeration was fist observed in the 1930's by Patterson & Cawood [36], which led to a series of independent experiments [37–40]. The interest in acoustic agglomeration was subsequently renewed in the 1980s with several theories been proposed and to estimate particle size growth [41–50]. In an acoustic field, particles are subject to a combination of different mechanisms that promote interactions. Among them, the orthokinetic and hydrodynamic interactions [45,51] are most widely recognised as the dominant mechanisms, while other effects such as acoustic streaming and turbulence further promotes the coagulation process. After collision, the particles are likely to adhere together due to meshing of irregular structures as well as inter-molecular attraction forces [52].

### 2.1 Orthokinetic mechanism

The orthokinetic mechanism refers to collisions between different sized particles located within a distance that is approximately equal to the displacement amplitude of the acoustic field and with their relative motion substantially parallel to the direction of vibration [40,53]. The acoustic field comprises of incident waves that are emitted directly from sound sources and scattered waves due to the presence of solid particles. The strength of the latter is strong in the case of large particles or high frequencies, and its effect on particle motion is important if separation distances between particles are small (high concentration) [29].

**Fig 2:**
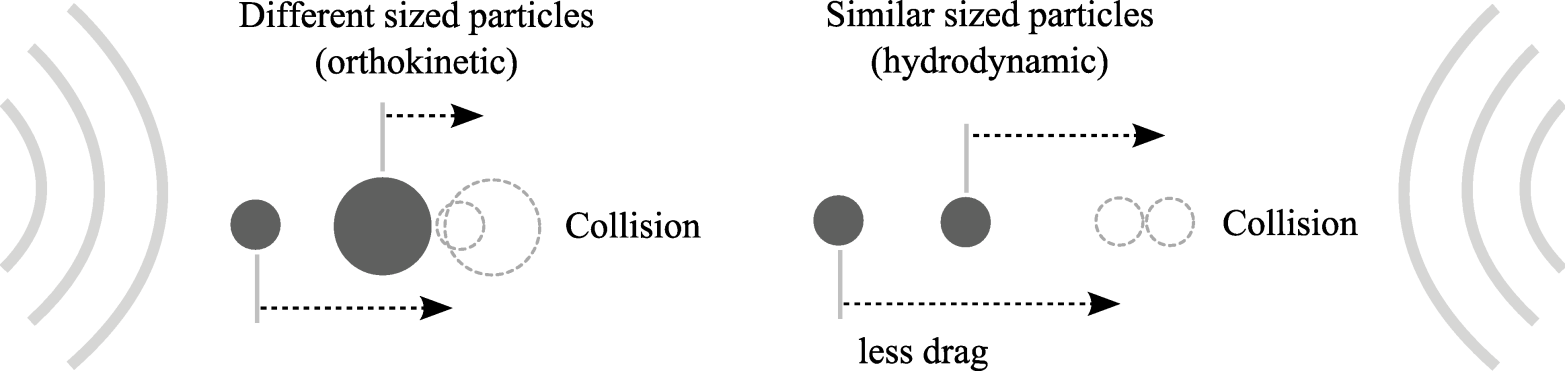
Orthokinetic and hydrodynamic mechanisms. Due to differential fluid and inertia forces, particles become entrained at different amplitudes and phase in the oscillations of an acoustic field. Consequently, the relative motions between the different sized particles result in collisions.

Due to differential fluid and inertia forces, particles become entrained at different amplitudes and phase in the oscillations of an acoustic field [25]. Smaller particles tend to follow the acoustic vibrations closely, while larger particles rarely move with acoustic waves due to their larger inertia [37,54–56]. Consequently, the relative motions between the different sized particles result in collisions, as shown in Fig 2. In general, particles of high density, large radius or the use of high frequency acoustic waves results in less entrainment.

### 2.2 Hydrodynamic mechanism

The orthokinetic model does not explain the observation of interactions between particles initially separated at distances much larger than the acoustic displacements and the agglomeration of particles of similar sizes [28,57]. In these instances, the mechanism for agglomeration can be explained by the hydrodynamic model. Hydrodynamic interaction refers to collisions caused by the viscous interaction between particles and their surrounding medium (air in the case of ACMV application), and can occur for particles that are separated at distances much larger than their acoustic displacement amplitudes. In general, two approaches to account for hydrodynamic forces have been proposed—mutual radiation pressure interaction and the acoustic wake effect.

Mutual radiation pressure interaction arises due to the nonlinear interaction between incident and scattered waves that imparts momentum to surrounding particles [53]. As a result, pressure gradients are created on different sides of the particle to give rise to a pattern of attraction and repulsion zones around the particle [54]. This constitutes the Bernoulli effect where two particles with line of centres oriented off-axis with respect to the acoustic velocity vector will experience attraction. In the case of on-axis orientation, repulsion will be observed [58–61].

Acoustic wake effect refers to the non-linear interaction of scattered waves and drag reduction experienced by a trailing particle travelling in the acoustic wake of a leading particle [62–68]. This causes the trailing particle to move at an accelerated speed towards the leading particle, resulting in collision and agglomeration, as shown in Fig 2. This effect is significant for particle agglomeration under high acoustic intensity and for particles of similar sizes. The acoustic wake effect causes rapid approach between particles with line of centres aligned parallel to the acoustic velocity vector and has a larger spatial influence over mutual radiation pressure interaction. It can thus be hypothesized [54,57] that at a certain particle separation distance, the forces due to mutual radiation and acoustic wake will counter-balance and the particles will remain closely spaced. In addition, the effects of gravity has a considerable effect on the particle trajectories under the influence of the acoustic wake [66,69].

The acoustic wake phenomenon has been confirmed through visualisations of monodispersed particles at high frequencies [54,70,71] and is significant for both polydispersed and monodispersed particles [72]. In addition, particle interaction due to acoustic wake increases with frequency but levels out at high frequencies, and has a non-linear dependence on acoustic intensity [73]. In general, the orthokinetic and hydrodynamic mechanisms are not completely independent and the increase in one may reduce the effect of the other [74].

### 2.3 Secondary effects

#### 2.3.1 Acoustics streaming

Acoustic streaming is a steady fluid flow formed by viscous attenuation of an acoustic wave. Depending on the mechanism behind the attenuation, streaming flows can vary in terms of the velocity, length scale and geometry [75–77]. The largest scale of acoustic streaming is known as Ecklart streaming that emanates from the acoustic source. As the wave propagates, acoustic energy is lost to the fluid at a rate proportional to the square of the frequency. As a result, acoustic pressure amplitude decreases with distance from the source and steady momentum flux is created. In the vicinity of a solid boundary, viscous dissipation into the boundary layer creates rotating streaming vortices known as Schlichting and Rayleigh steaming in the inner and outer boundary layers, respectively. This creation of streaming flows within the acoustic chamber can promote inertial impaction and subsequent agglomeration and deposition of PM [78–81].

#### 2.3.2 Turbulence

At high acoustic intensities (above 160 dB), acoustically induced turbulence is also generated and promotes particle collisions [18–20,82,83]. There are in general two mechanisms associated with turbulence agglomeration. In turbulent diffusion, particles chaotically collide as a result of differential velocities caused by the spatial inhomogeneity of a turbulent flow, whereas in turbulent inertia collisions, particles collide due to relative motions from their inability to follow rapid turbulent motions. Acoustically induced turbulence have shown to be little affected by variations in the acoustic frequency and the increase in agglomeration rate due to turbulence would eventually saturate as the sizes of turbulent eddies become smaller at higher acoustic intensities that reduces particle interactions [82,84].

#### 2.3.3 Ambient conditions

Ambient conditions have indirect effects on particle agglomeration. Particularly, ambient pressure changes the density, kinematic viscosity and acoustic impedance of the fluid medium. Higher pressure increases density and thus imposes larger impedance to particle oscillations [24,82]. Humidity also plays a role as water droplets act as collectors for fine PM [85]. Another mechanism responsible for particle collision is Brownian motion (random motion of particles) and is important for small particles (sub-micron particles) of high concentrations [86–88].

## 3. Summary of previous studies on acoustic agglomeration

A summary of previous experimental investigations into the acoustic agglomeration phenomenon and performances is documented in Table 2. Most of the works focused on applications in industrial emissions and the large variability in the reported agglomeration rates originates from the agglomeration environment in which the experiments were conducted. In particular, the particle characteristics and acoustic properties play critical roles in determining the resulting agglomeration performances. Other parameters such as the addition of seed particles, humidity and turbulence play secondary roles in the particle coalescence process.

**Table 2:** Summary of relevant experimental works in acoustic agglomeration with reported performances.

Reference	Particle type	Particle size and distribution	Particle number conc. (m^-3^)	Frequency (kHz)	Intensity (dB)	Residence time	Performance
Boulaud et al. [89]	DOP	Monodisperse	-	0.54 & 1.02	140 to 160	3.8 & 8.6	Shifted mean from 1.5 μm to 4.5 μm & shifted σ from 1.75 to 5
de Sarabia and Gallego-Juárez [21]	Black smoke	Polydisperse	-	20.4	161	5	Shifted mean particle size from sub-micron to above 5 μm
Gallego-Juárez et al. [90]	Fly ash	Polydisperse	10^11^	10 & 20	152	2	Number conc. of micron & sub-micron particles reduced by 70% & 30%, respectively
Capéran et al. [91]	Fly ash	Polydisperse	10^11^	21	143	30	Initial agglomeration rate was 3.3 times that due to Brownian
Hoffmann et al. [74]	Fly ash & limestone as sorbent	0.5 μm (fly ash) 88 μm (limestone)	-	0.044	160	1.0 to 3.0	Mass conc. of particles < 11 μm reduced by 23%
Gallego-Juárez et al. [92]	Fly ash	Monodisperse, 0.5 μm	-	10 & 20	145 to 165	2.0	Number conc. of micron & sub-micron particles reduced by 42% & 39%, respectively
Shuster et al. [93]	Incense	Polydisperse	10^13^	0.0433	> 160	30 to 50	Particles shifted from sub-micron to micron size in weak periodic shock waves
Moldavsky et al. [94]	Arizona test dust	Polydisperse	-	0.05 to 1	110 to 130	-	Fibrous filter operating time can be extended 2 to 10 times
Liu et al. [95]	Fly ash	Tri-modal, 0.1, 0.76 & 1.95 μm	10^11^	1.4	147	4.0	Number conc. of PM_2.5_ reduced by 75.6%
Liu et al. [96]	Fly ash	Bi-modal, 0.071 & 0.76 μm	10^11^	1.4	150	4.0	Number conc. reduced by 75.3%
de Sarabia et al. [85]	Diesel exhaust	Quasi- monodisperse with mode 0.06 μm to 0.1 μm	10^14^	21	151	2.7	Number conc. without & with humidity reduced by 25% & 56%, respectively
Noorpoor et al. [97]	DOP	Monodisperse, 0.26 μm	10^12^	0.83	145	-	Efficiency of precipitation increased by 43% to 93% depending on residence time
Guo et al. [98]	Fly ash	Polydisperse	-	1.416	120	-	Mass conc. of particles 3.3 μm reduced around 35% from combined acoustics, 23 ms^-1^ jet gas & seed particles of 150 μm to 250 μm
Sun et al. [99]	Fly ash	Polydisperse	-	1.416	128	-	Mass conc. of particles < 2 μm reduced from 40% to 10% with combined acoustics & 25.5 ms^-1^ jet gas
Yuen et al. [78]	Polystyrene spheres	Monodisperse, 0.3 μm to 0.6 μm	-	30	150	-	Number conc. of micron & sub-micron particles reduced by 25% & 12%, respectively. This was increased to 32% & 20% with acoustic streaming
Zhou et al. [100]	Fly ash	Polydisperse	10^11^	1.4	142	4.4	Number conc. reduced by 35%
Yan et al. [101]	Coal dust & SDS as Seed droplet	Unimodal, 0.3 μm (dust), 20 μm (SDS)	10^12^ (dust), 10^9^ (SDS)	2.0	150	-	Number conc. of sub-micron particles reduced by 56.7%
Ng et al. [102]	Arizona test dust	Polydisperse	10^10^	6.4	140	4.0	Number conc. of particles 0.4 μm to 0.5 μm reduced by 16%

All particle sizes are given by their diameters. Conc, concentration; DOP, dispersed oil particulate; SDS, sodium dodecyl sulfonic salt wetting agent; σ, standard deviation.

### 3.1 Particle characteristics

Particle characteristics such as size distribution and concentration affects the dominant mechanism in the agglomeration process and hence the observed particle interactions.

#### 3.1.1 Particle size distribution

Among the different mechanisms for acoustics agglomeration, the hydrodynamic mechanism exists regardless of the particle size distribution [61]. However, its significance in the agglomeration process depends on whether the particle size distribution is of monodispersed, bi-modal or polydispersed characteristics. For monodispersed particles, the hydrodynamic mechanism is dominant in promoting particle collisions as all particles are entrained at the same amplitude and phase in the oscillations of the acoustic field. The dominance of the hydrodynamic mechanism (specifically the acoustic wake effect) in stimulating monodispersed particle agglomeration was experimentally visualised by Hoffmann and Koopmann [54] using monodispersed glass microspheres. The microspheres were shown to remain in a captive zone due to the counterbalance of the acoustic wake and mutual radiation pressure interference at a frequency of 0.6 kHz. Above this frequency (0.8 to 0.9 kHz), the particles are seen to converge as an indication of the dominance of the acoustic wake effect.

On the other hand, for particles of bi-modal characteristics, particle agglomeration is mainly driven by the orthokinetic mechanism due to differences in entrainment of the different sized particles. This type of particle distribution is often found in emissions from industrial processes. For instance, in applications where sorbent materials (e.g., limestone) are used to remove sodium dioxide, these materials can also act as collectors (collision partners) under the influence of an acoustic field and further encourage particle interactions by reducing distances between particles through higher concentrations. For polydispersed particles, both orthokinetic and hydrodynamic mechanisms play a significant part in the agglomeration process [29,69,103,104].

#### 3.1.2 Particle concentration

A larger particle number concentration signifies more particles within a specific volume that enhances the probability of collision and agglomeration [79]. In Capéran et al. [105], particle agglomeration rate increased almost linearly with particle number concentration until a maximum asymptotic value is reached at a concentration of around 3 × 10^12^ m^-3^. On the other hand, at low concentrations, using fly ash of tri-modal distribution, agglomeration efficiency falls off steeply as concentration drops below 1.7 × 10^11^ m^-3^ [74]. The orthokinetic mechanism may be less dominant due to larger spacing between particles at low concentrations [59] and hydrodynamic interactions could play a major role. However, it has also been shown that small or large concentrations may not be favourable for acoustic agglomeration and there is a range of concentrations for optimal particle growth [21].

### 3.2 Acoustic properties

The fundamental driver in acoustic agglomeration is the property of the acoustic signal. Most importantly, the acoustic frequency, intensity and residence time are dominating factors in affecting particle entrainment, rate of agglomeration and exposure to acoustic field, respectively.

#### 3.2.1 Frequency

**Fig 3:**
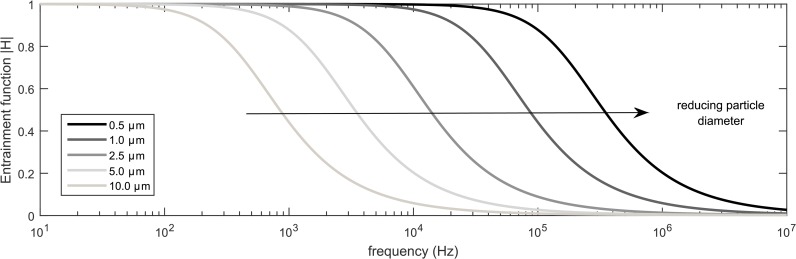
Effects of frequency and particle diameter on entrainment. A small value of entrainment function |H| indicates that the particle is relatively motionless in the acoustic field and a value close to 1 means that the particle is fully entrained and oscillates with the gas medium.

The degree of particle entrainment in an acoustic field is dependent on the acoustic frequency [54,55]. In general, lower frequencies in the audible range are effective for the agglomeration of particles in the micron and sub-micron range, whereas higher frequencies in the ultrasonic range perform better for particles in the sub-micron range [92]. This is evident in Fig 3 where the frequency threshold for particle entrainment of smaller particles is larger [96]. At 20 kHz, even though micron particles (10 μm diameter) remain almost stationary, sub-micron particles (0.5 μm diameter) are still entrained in the acoustic wave. In other words, particles of larger diameters and higher densities will require lower acoustic frequencies [40].

**Fig 4:**
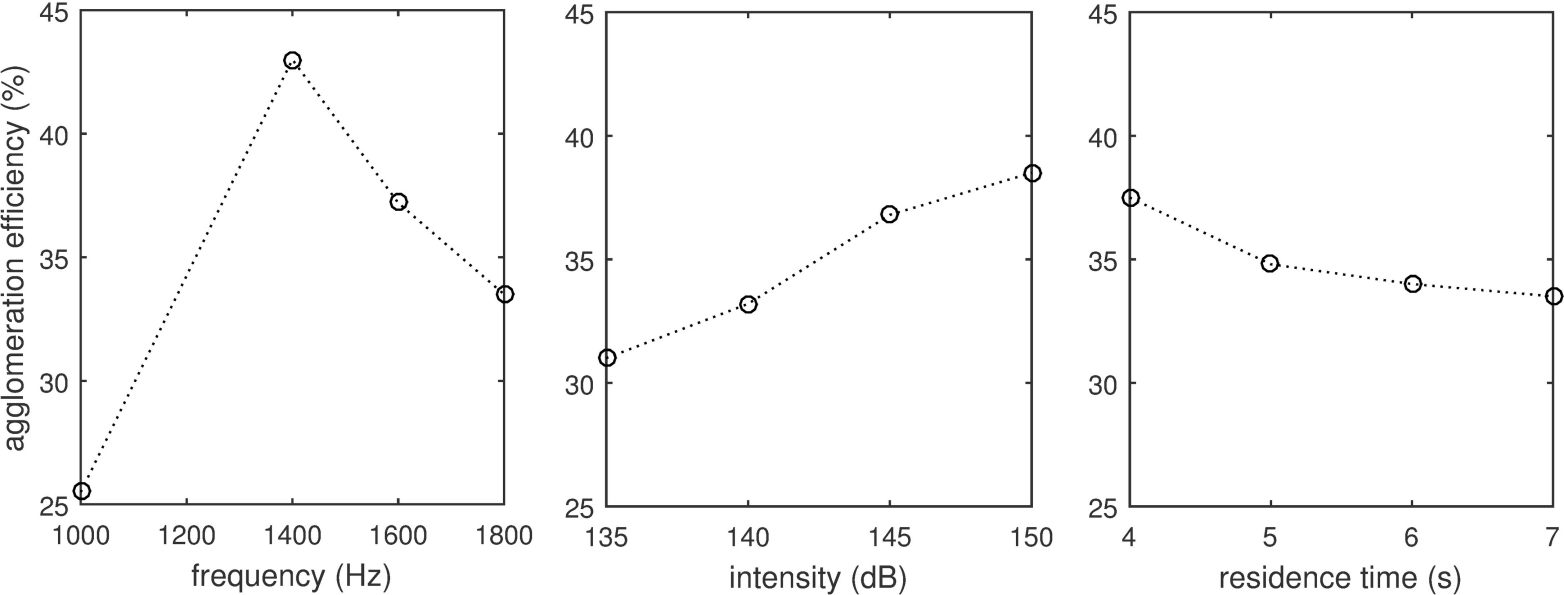
Sound parameters on acoustics agglomeration. Effect of frequency, intensity and residence time on agglomeration efficiency. (Reproduced from Liu et al. [96] for fly ash with bi-modal characteristics).

It has been demonstrated that for specific particle size distributions, there exists a frequency that optimises the coalescence process, which is inversely related to the particle relaxation time for size distributions that are not too wide [45]. The particle relaxation time expresses the time for the particle to react to an external excitation. The existence of the optimal frequency was demonstrated by Liu et al. [96] using fly ash of bi-modal characteristics (with peaks at 0.071 μm, 0.76 μm and concentration of 10^11^ m^-3^) where the dominant mechanism was orthokinetic. The optimal frequency was 1.4 kHz as shown in Fig 4. In addition, the optimal frequency for particle agglomeration was found to decrease with acoustic intensity [69,95] as higher SPL promotes rapid agglomeration of particles to larger clusters that have different (lower) optimum frequencies. As suggested in previous studies [40,92], a multi-stage acoustic agglomeration scheme can be adopted such that frequency can be altered as particles agglomerate along the path of fluid flow. To remove the frequency dependence, progressive saw-tooth wave can also be considered [106].

#### 3.2.2 Intensity

The rate of orthokinetic and hydrodynamic agglomerations, streaming as well as onset of acoustic turbulence are dependent on the acoustic intensity [18,82,107]. In general, an almost linear relationship exists between the initial acoustic agglomeration rate and the applied acoustic power [105], which can also be observed in Fig 4 where agglomeration efficiency can be elevated by more than 50% when SPL was increased from 120 dB to 157 dB [101]. Although most studies favoured high levels of SPL, it has been shown that particle diameter can increase by one order of magnitude [47] at low SPL of 100 dB to 120 dB under large residence times (at least 20 seconds). Also, having more transducers help to reduce particle concentration due to the increased spatial occupation of the acoustic agglomeration zone [108].

#### 3.2.3 Residence time

Residence time determines the amount of exposure to the acoustic field and has an almost linear relationship with particle size growth [21,74]. However, for each specific acoustic frequency, there is a limit to the residence time for which effective particle agglomeration can be maintained. With an increase in residence time, particles agglomerate to larger sizes and adopt a new spectral distribution. As a result, particle entrainment is lowered and new acoustic parameters will be necessary if further agglomeration is required. In Hoffmann et al. [74], it has been shown that the effectiveness of particle agglomeration slows down beyond 3.5 seconds.

As particles agglomerate, the remaining and agglomerated particles become more widely spaced (i.e. lower particle number concentration) and opportunities are given for breaking-up of the agglomerated particles. This was also observed in Liu et al. [96] in Fig 4 where agglomeration efficiency decreased with longer residence times. Having a long residence time may otherwise also be difficult to maintain as it is determined by the local air flow velocity, requiring a large agglomeration chamber or a long array of acoustic devices.

### 3.3 Other influencing parameters

The introduction of seed particles can promote particle agglomeration as they act as collision partners for fine PM within the fluid medium. Typically, seed particles have sizes larger than 15 μm and are less entrained than fine PM, resulting in relative motions that promote collisions [109,110]. The presence of seed particles enable agglomeration even at low acoustic intensities, which translates to lower energy consumption [111]. However, there is an optimum amount of seed particles for maximum increase in efficiency and excessive amounts would limit the agglomeration process. There is also the challenge of dispensing the solid particles evenly in the fluid medium.

Apart from solid seed particles, water droplets can also be introduced to promote agglomeration [24,109,112]. The effect of humidity on agglomeration was investigated by de Sarabia et al. [85,113] where an increase in moisture content reduced particle number concentration by 56% due to particle condensation. With acoustics alone, the particle number concentration was only reduced by 25%. On the other hand, water droplets have large surface tension where fine particles (mostly hydrophobic) do not adhere or penetrate [114–116]. A solution is to change the surface property of particles from hydrophobic to hydrophilic by introducing surface active agents. These surface agents increase the wettability of fine particles such that they can penetrate into the droplets easily (through capillary action) and thus promote agglomeration after collision in an irreversible process [101].

Acoustic agglomeration is little affected by elevated temperatures [74], and gravitational settlement was found to be negligible compared to acoustic agglomeration [42]. Likewise, wall deposition has a small effect on acoustic agglomeration as the particles deposited are normally the larger ones. These larger particles, if left suspended in the fluid, can act as capturers for smaller particles [52]. The effect of turbulence on acoustic agglomeration is less conclusive. Some have reported little improvement in particle agglomeration in the presence of turbulence [20], while others have observed dominating effects on the overall acoustic agglomeration rate [18,82].

Agglomeration was shown to be more effective under standing wave conditions [82,105,117] as the acoustic drift towards standing nodes introduces spatial non-uniformity of aerosol concentrations. As particle concentration plays a critical role in particle interactions, the increase in local concentration at the nodes promotes particle agglomeration [118]. It has been shown that by using soot from the burning of polystyrene foam in a closed chamber with standing waves, soot disks (regions of high concentrations) are formed at stable positions within the chamber, which resulted in particle agglomeration that is eight times faster than gravity and diffusion alone [119]. Despite claims of better performances with standing waves, a quantitative comparison between the effectiveness of travelling and stationary waves in promoting particle agglomeration is still lacking [71].

The diverse operating conditions in the experimental studies contribute significantly to the variability of reported performances and more information (residence time, humidity, etc.) will be necessary for a comprehensive understanding of the agglomeration phenomenon under different set-ups. For instance, the intensity of acoustic sources varies with frequency and there is an optimum frequency for SPL to be maximised. Hence it was not clear if the higher performances reported at higher or lower frequencies is due to the non-linearity in the acoustic source. Also, it was not clear if ambient conditions in the different experimental set-ups played critical roles in the reported performances [85]. Likewise, the size and configuration of the acoustic chamber may have an effect on the observed agglomeration process and this was little investigated. In general, results on agglomeration performances and the determination of optimal acoustic frequencies, intensities or residence times are specific to the particles and operating conditions used in the experiments. For different applications, such as in industrial or ACMV context, the particles and operating conditions that are simulated will have to be specific to the relevant context and the results on parametric studies are also expected to draw different conclusions.

The non-uniformity of particles inserted into the agglomeration chambers may contribute to the observed changes in particle mass, number concentration and sizes, thus rendering the reported performance from acoustic agglomeration to be higher or lower than it actually was. It was not clear in most of the experiments on the method of particle insertion, duration of experiments and how many times [85] the experiments were repeated to average out errors from particle insertion. Even for commercial particle dispensers, the uniformity in time and spectral distribution of dispersed particles is a challenge [120]. Lastly, instrumentation bias may be a concern and the experimental results will benefit by further reporting on benchmark instrumentation validations against known particle size distributions or other instruments.

### 3.4 Application to ACMV systems

PM is a common indoor air pollutant composed of a mixture of suspended solid particles and liquid droplets of varying sizes. PM_10_ (coarse particles of 2.5 μm to 10 μm in diameter) could originate from sources such as roads and constructions. On the other hand, PM_2.5_ (fine particles of less than 2.5 μm) and UFP (ultrafine particles of less than 0.1 μm) could originate from combustion, industrial, mobile emissions and can have detrimental health effects [121,122]. These outdoor PM are also capable of penetrating indoors where there are already existing PM sources from cooking [123], dusting [124], ACMV systems [125], smoking [123,126], resuspension [127], etc. Bioaerosols refers to suspended airborne particles that contain living organisms or are released by those organisms in both indoor and outdoor environments [128]. The size of bioaerosols range from less than 0.1 μm to around 100 μm in aerodynamic diameter [129] and they include virus, bacteria, fungal spores, pollen and house dust mites that have adverse effects on human health [130–140].

**Fig 5:**
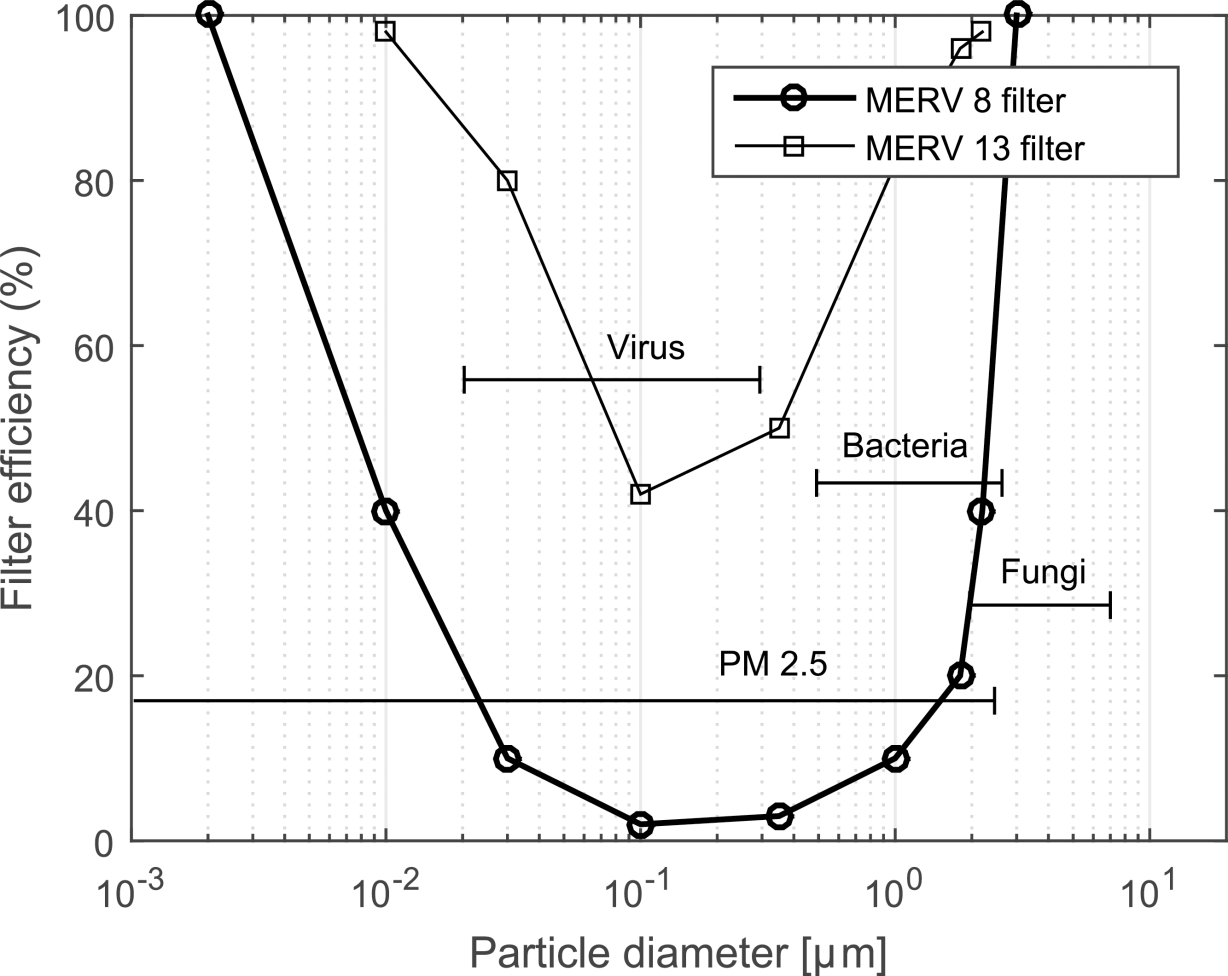
Filtration efficiency of MERV 8 and 13 filters. Relationship between filter efficiency and particle size [33], including the size distributions of virus [152], bacteria [153,154] and fungi [151,155].

Apart from dilution, filtration is the primary mechanism for the removal of PM and bioaerosol in ACMV systems [141,142]. However, as shown in Fig 5, the filtration efficiency of typical filters varies according to particle size where capture rates for smaller (< 0.01 μm) and larger PM (> 2 μm) are higher than that for intermediate sized particles (0.01 μm to 2 μm). Smaller particles are mainly captured by diffusion (Brownian motion) while larger particles are mainly filtered through interception/inertial impaction [143]. In the intermediate size range, however, neither of the mechanisms could play a significant role [144]. Meanwhile, these intermediate particles constitute a large fraction of airborne PM both indoors [145,146] and outdoors [147]. As a result, modern building codes and guidelines tend to heighten requirements to use high efficiency filters to remove particles in the intermediate size range [148–150]. For instance, in the Singapore code of practice for indoor air quality in buildings with ACMV systems [151], a double stage filtration for controlling of indoor air quality has been recommended. This requires a filter of MERV (minimum efficiency reporting value) rating 6 or higher and a secondary filter of MERV rating 13 or higher to be installed.

However, high efficiency filters create large pressure drops that necessitate higher fan energy consumption under the same airflow rate [156–163]. Furthermore, for existing buildings under retrofitting to meet the modern indoor air quality standards, heightened filtration requirements often lead to extra costs to upgrade the air distribution system. To overcome the fan energy demands and extra cost of system upgrading, a solution would be to find methods to allow for lower removal efficiency filters to be used without sacrificing on particle removal efficiencies. Acoustic agglomeration could be one of the methods. With the extensive use of ACMV systems in cities, the accumulated energy savings in overcoming a lower pressure drop across filters could be substantial. Furthermore, many existing ACMV systems are designed for low grade filters and such pre-conditioning mechanism can avoid the cost for booster fan replacements that accompany high efficiency filters. Apart from particle agglomeration prior to filtration, acoustics can also be used within the same control volume together with filters to increase the incidence of impactions of particles on the fibrous media [164] and even extending the operating life of filters [94,165,166].

## 4. Experimental feasibility study

The hypothesis of the current experimental study is that airborne fine PM could be pre-conditioned by means of acoustic agglomeration to form larger clusters that can subsequently be removed more easily by air filtration in ACMV systems. As a feasibility study, experiments were carried out in a small-scale wind-tunnel with the objective of investigating the potential improvement in filtration efficiencies of air filters resulting from acoustic pre-conditioning of airborne PM.

From the review of acoustics agglomeration in previous sections, it is determined that at least 140 dB is required with sufficient residence time for any changes in particle size distribution to be observed and standing waves are preferred for particle drift towards nodes. These are parameters considered for the feasibility study. Other influencing parameters such as the addition of seed particles, turbulence, humidity, etc. are not explored in this preliminary investigation, but will be considered in future studies.

### 4.1 Experimental set-up

**Fig 6:**
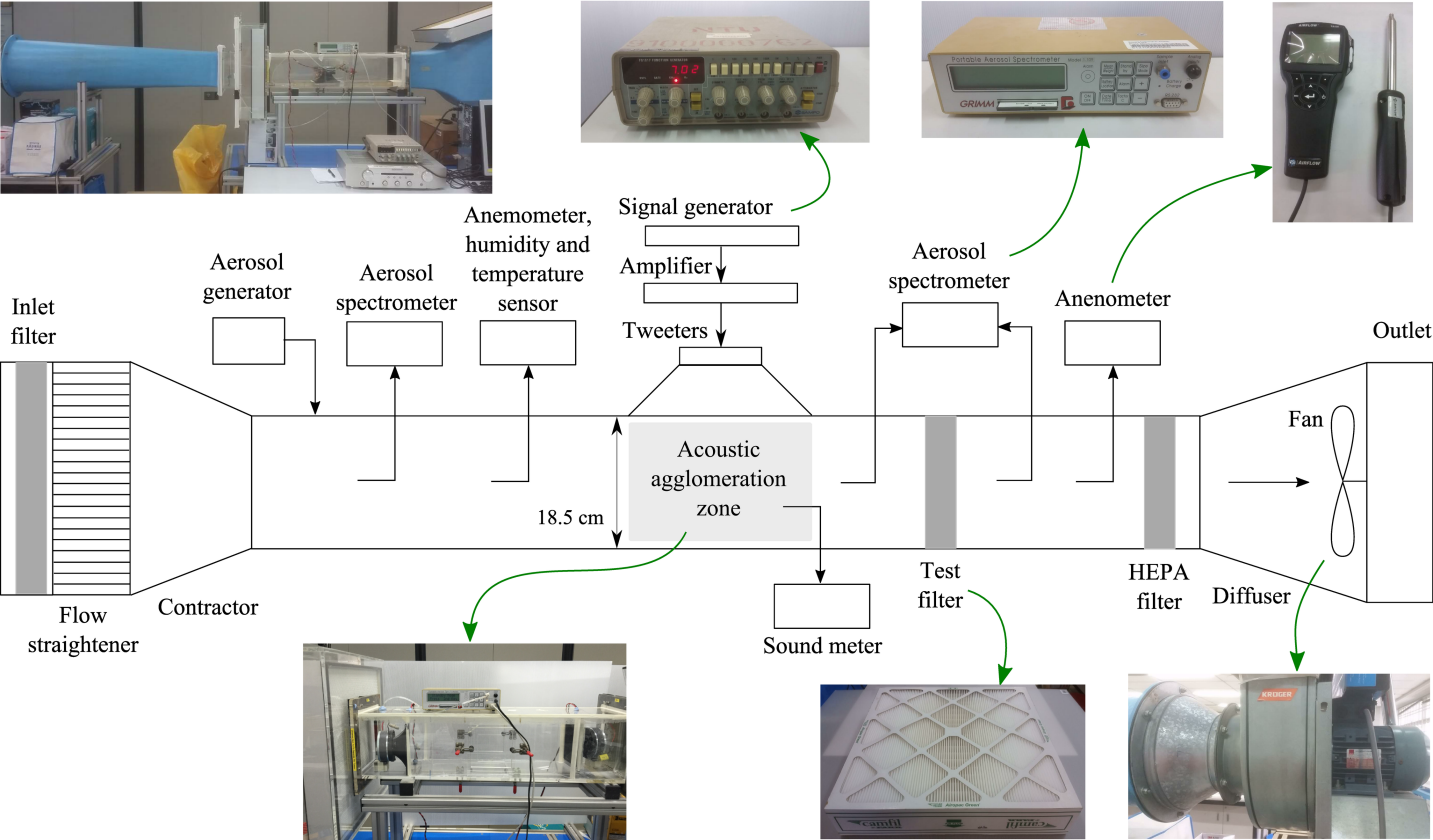
Schematic of experimental set-up. Experimental set-up simulating travelling airborne PM in an open-loop, draw-through wind tunnel (resembling a ventilation duct) with acoustic agglomeration pre-conditioning prior to a test filter that is typically used in ACMV systems.

The experimental set-up simulates travelling airborne PM in an open-loop, draw-through wind tunnel (resembling a ventilation duct) with acoustic agglomeration pre-conditioning prior to a test filter that is typically used in ACMV systems. As shown in Fig 6, the main section of the set-up consists of a wind tunnel, agglomeration zone, test filter and fan at the end of the tunnel. The wind tunnel is designed with a contraction at the inlet, a diffuser at the outlet, and a metre-long test section with internal dimensions of 18.5 cm by 18.5 cm. The airflow is induced by a fan (Kruger Engineering, FSA200/CM) at the end of the tunnel for which the fan speed is controlled by a frequency inverter. Air flow velocity in the test section is measured using a thermal anemometer (TSI TA430), which is also capable of temperature measurements. These measurements are monitored before the agglomeration zone and after the test filter.

The test particles with primary composition of silica (ISO 12103–1 A1 ultrafine test dust) are inserted upstream of the agglomeration zone. The polydispersed characteristics of the test particles can be representative of airborne PMs typically found in ACMV systems. The particle count in the tunnel before and after the test filters are measured using an aerosol spectrometer (GRIMM, 1.109), which is based on the light scattering principle [167,168]. The spectrometer measures particles from 0.25 μm to 32 μm in 31 size channels and is user-interfaced with a computer running GRIMM spectrometer software version 4–0. The sampling points are prior to the agglomeration zone, and before and after the test filter. The acoustic agglomeration zone consists of a tweeter, an amplifier and a signal generator. Experiments are conducted with two air filters, rated MERV 11 and 13 (Airopac^®^ Green model 3GP-20204-60 & 3GP-20204-90) at the airflow rate of 0.65 m^3^s^-1^. The pressure drop across the two new test filters are documented as 75 Pa and 140 Pa, respectively [157]. At the end of the test section, an exit HEPA filter is used to prevent PM contamination of the laboratory housing the experimental set-up.

The experiments are conducted to investigate the PM concentrations after filtration with and without pre-treatment of acoustic agglomeration. For each set of experiment, 20 samples are taken at each sampling point and averaged to reduce any effect of inconsistency in aerosol generation. To a large extent, the characteristics of particles depend on the method of generation and there is a wealth of literature on state-of-the-art methods for mono- and poly-dispersed particle generation [169–174]. Particle generation can be classified into wet [175,176] or dry forms [120,177] and in the current study, a mixer type system was adopted where dry polydispersed particles are released in bursts of 3 seconds interval into a mixing chamber before being fed into the wind tunnel test section [120]. The sound level is monitored throughout each experimental run and the sampling time for PM is 6 seconds. The number of particles loss through wall deposition is assumed to be negligible compared to the total particle concentration [105]. Under low acoustic intensity, the effects of turbulence and secondary non-linear effects can also be assumed to be small compared to the acoustic agglomeration phenomenon [42]. At the start of each experimental run, the flow speed and temperature are recorded. At the end of each experimental run, the test section is ventilated and flushed of PM that may have deposited on the walls.

The lower efficiency filter is first installed in the test section and the air flow is adjusted using the frequency inverter to achieve a flow speed of 0.15 ms^-1^, equivalent to a particle residence time of 4.0 seconds in the agglomeration zone. PM concentration is first measured without acoustics before the agglomeration zone and after the test filter. Subsequently, acoustics is turned on and PM concentration is then sampled before the agglomeration zone as well as before and after the test filter. The procedure is then repeated with the higher efficiency filter.

### 4.2 Experimental results

**Fig 7:**
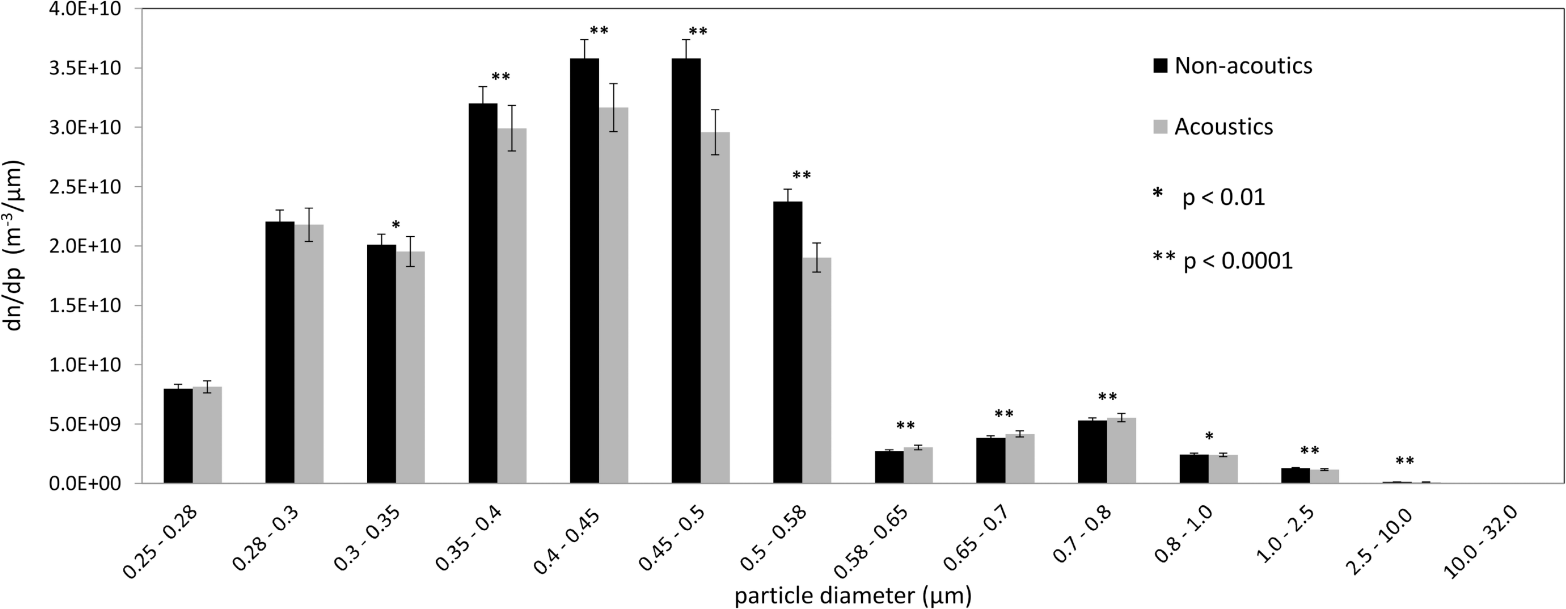
Experimental results on the effect of acoustic agglomeration on particle size concentration. In the smaller size range, number concentration of particles in the range of 0.4 μm to 0.5 μm in diameter is reduced by almost 16%. In the intermediate size range of around 1 μm to 2.5 μm, we observe another drop in number concentration of 10%.

The effect of acoustics on PM agglomeration is first determined by sampling before and after the agglomeration zone. This is shown in Fig 7 with a 4% and 6% error for the experimental results with and without acoustics, respectively. The frequency of 6.4 kHz was chosen based on a frequency sweep and a maximum intensity of 140 dB was attained using this frequency in the current acoustic set-up. Two distinct regions of agglomeration were observed. In the smaller size range, number concentration of particles in the range of 0.4 μm to 0.5 μm in diameter is reduced by almost 16%, with an accompanying 8% increase in number concentration in the 0.6 μm to 0.8 μm range for which the particles could have agglomerated. In the intermediate size range of around 1 μm to 2.5 μm, we observe another drop in number concentration of 10%, which has agglomerated to larger particle sizes that are of low numbers and beyond the interest of this study as they can be easily captured by conventional filters. The difference in concentration with and without acoustics is significant with p < 0.01 for particles greater than 0.3 μm in diameter in a t-test. The agglomeration phenomenon is frequency dependent and it should be noted that agglomeration in the ultrasonic range may exhibit slightly different performances, which is a subject for further experimental investigations with a different acoustic system.

**Fig 8:**
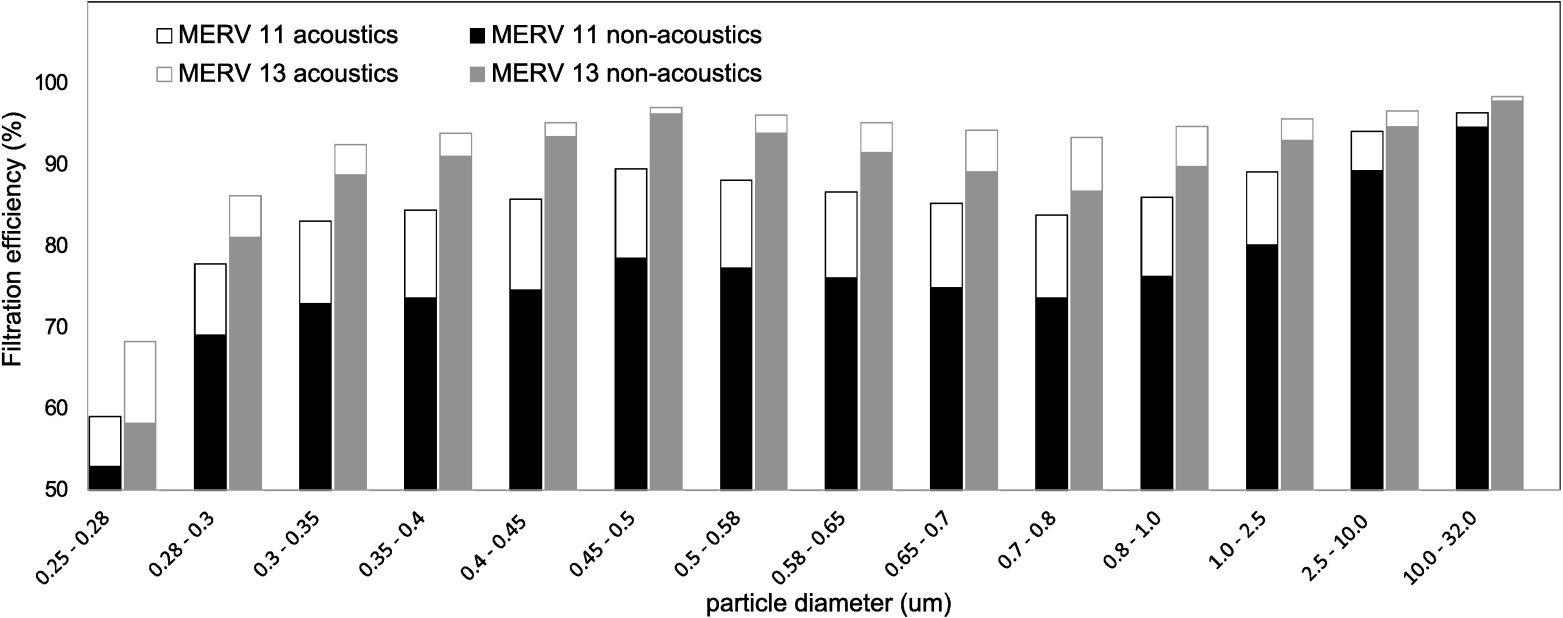
Experimental results on the filtration efficiencies of MERV 11 and 13 filters with and without acoustic agglomeration pre-conditioning. Filtration efficiency is expressed as the percentage drop in particle number concentration before the agglomeration zone and after the filter. With acoustic pre-conditioning, the filtration efficiency of the MERV 11 filter is increased by about 10%, bringing its filtration efficiency closer to that of the MERV 13 filter without acoustic pre-conditioning.

The filtration efficiencies of the lower and higher efficiency filters (rated MERV 11 and 13, respectively) enhanced with acoustic pre-conditioning are investigated by sampling before the agglomeration zone and after the filter. The change in particle concentration with filter is shown in Fig 8 where the filtration efficiency is expressed as the percentage drop in particle number concentration before the agglomeration zone and after the filter with the relationship

$$\mathrm{filtration}\;\mathrm{efficiency}=\left(1-\frac{n_{o,af}}{n_{o,ba}}\right)\times 100\%,$$ (1)

where *n*_*o*,*af*_ is the number concentration after filter and *n*_*o*,*ba*_ is the number concentration before agglomeration zone.

The filters exhibit lower efficiencies for fine PM below 0.3 μm and show a slight dip in the 0.6 μm to 0.8 μm range. With acoustic pre-conditioning, the filtration efficiency of the MERV 11 filter is increased by about 10%, bringing its filtration efficiency closer to that of the MERV 13 filter without acoustic pre-conditioning. The agglomeration process improved the filtration efficiency of the MERV 11 filter for PM_2.5_ from 73% to 83%. With the MERV 11 and 13 filters having documented pressure drops of 75 Pa and 140 Pa, respectively, the use of the former as a replacement to the latter can bring about significant fan energy savings without compromising much on filtration efficiencies. When acoustic pre-conditioning is applied to the MERV 13 filter, its filtration efficiency for PM_2.5_ was improved from 88% to 92%.

Quantifying potential energy savings in the ACMV context, the fan power for a typical ventilation system can be expressed using the relationship [9,150]

$$\mathrm{Fan}\;\mathrm{power}=\frac{\mathrm{Airflow}\;\mathrm{rate}\times \mathrm{Total}\;\mathrm{pressure}\;\mathrm{drop}}{\mathrm{Overall}\;\mathrm{fan}\;\mathrm{efficiency}}$$ (2)

where the total pressure drop is across the whole air distribution system. With the replacement of a MERV 13 filter with a MERV 11 filter, the savings in pressure drop is 65 Pa. The typical overall fan efficiency (including motor, power transfer and aerodynamic efficiencies) in air handling units (AHU) range between 0.15 to 0.45 from residential to commercial buildings [12,178]. Assuming a fan efficiency of 0.15 in Eq (2), the fan power savings from the filter replacement is around 280 W for the factory tested airflow rate of 0.65 m^3^s^-1^, as shown in Table 3. These power savings are considerably larger than the power consumed by the acoustics system of 30 W used in the feasibility study. This, as a whole, demonstrates potential energy savings from the combined acoustic-enhanced filtration system with little compromise on particle removal efficiency.

**Table 3:** Filter properties [157] and computed fan power to overcome filter pressure drop in Eq (2), assuming an overall fan efficiency of 0.15.

****Filter****	****Dimension (m)****	****Air flow rate (m**^**3**^**/s)****	****Pressure drop (Pa)****	****Fan power (W)****
MERV 11	0.49 x 0.49 x 0.1	0.65	75	323
MERV 13			140	603

### 4.3 Discussions and challenges from feasibility study

The feasibility study indicates the possible enhancement of filter efficiency through acoustic agglomeration and identifies further scope of research in this area. It has been shown that lower efficiency filters with acoustic pre-conditioning can be used to replace higher efficiency filters without compromising on PM removal efficiencies. However, several challenges remain before they can be widely implemented on ACMV systems:

- Particle number concentration in the feasibility study is in the order of 10^10^ m^-3^ and the number of particles in the range of 0.4 μm to 0.5 μm in diameter is reduced by 16%. This is still some distance away from the average 40% reduction as demonstrated in earlier studies with higher concentrations. Potential enhancements could be in the form of a multi-stage agglomeration chamber of different frequencies along the path of particle fluid flow or addition of seed particles as collision partners. In addition, despite the numerous studies on agglomeration of PM (solid and liquid), there has been a lack of studies for bioaerosols where the effects of acoustics might not only be in particle agglomeration, but could also affect the viability/survival of them.
- Despite the potential of acoustic pre-conditioning in the enhancement of filter efficiency, the energy consumption for continual usage of acoustic devices needs to be considered and to offset any energy savings that are derived from using lower efficiency filters in ACMV systems [161,163]. Particularly, in the tropics where ACMV systems are heavily utilised, the acoustic system may have to be switched on for prolonged durations or even round the clock. Given the requirement of high acoustic intensity (> 140 dB) for agglomeration to occur within short residence times, the energy consumption by the acoustic system could be considerable. As such, optimisation studies will be necessary to identify specific conditions necessary for efficient particle agglomeration in ACMV systems with little compromise on power requirements. In addition, scheduling of the acoustic system may be able to save cost by using sensors to activate the acoustic system only above a user-defined threshold in PM concentration. For instance, during haze or when indoor activities such as cooking or cleaning are carried out, acoustic-enhanced filtration can suppress the temporary increase in fine airborne PM concentration, which would otherwise bypass the filtration process. Such an active device will also enable tuning of acoustic parameters for optimal agglomeration rate.
- In order for acoustics agglomeration to be effective, high sound pressure levels will be required and in the feasibility study, 140 dB at 6.4 kHz was used and this causes noise problem that needs to be addressed [179]. Solutions to mitigate the noise problem include the use of passive or active sound attenuation mechanisms. The former relies on the use of sound absorbing material such as foam in the lining of ducts [180] while the latter on actively controlled secondary acoustic sources to cancel the primary acoustic wave [181,182]. Additionally, as shown in Table 2, increasing the frequency to ultrasound is also able to deliver as good particle agglomeration efficiency as compared to the audible range. These are scopes for further investigations as part of an ongoing research work. Nonetheless, it should be noted that in commercial and industrial buildings, the AHU are often situated in dedicated machine rooms that are away from occupants or on rooftops where the effects of noise on building occupants can be minimised.
- For acoustic pre-conditioning to be implemented on existing ACMV systems, modifications to existing ducts are necessary. The modifications will need to accommodate the acoustic system and also an agglomeration zone with sufficient residence time at a position just prior to filtration devices. A longer acoustic residence time promotes particle agglomeration and could be a challenge in ACMV ducts where the flow speed is variable according to the ventilation loads. A possible solution may be to install a long array of acoustic sources or to enlarge the cross-section of the duct in the agglomeration zone in order to lower flow speed and hence increase residence time. Nonetheless, longer residences time also leads to changes in the particle size distribution [96,97] that requires fine-tuning of the acoustic conditions (intensity and frequency).

## 5. Conclusion

Acoustic pre-conditioning could be a solution to promote particle coagulation into larger agglomerates that can be better captured by air filters in ACMV systems. Studies reported in the literature have indicated that high acoustic intensities (> 140 dB) and concentrations are required for any observed agglomeration. Additionally, the optimal frequency is highly variable with particle size, distribution, concentration as well as acoustic intensity. Having longer acoustic residence time may also not necessarily be beneficial to particle agglomeration as the spectral distribution and concentration of particles would have changed. Other parameters such as turbulence, seed particles, streaming and humidity can further enhance the agglomeration process. However, the variability in ambient conditions, acoustics chamber set-up, sensitivity of acoustic devices and particle insertion methods on the observed agglomeration rates would require further examination.

Building on the previous findings, the application of acoustics agglomeration in ACMV systems is demonstrated through an experimental study on the feasibility to enhance the filtration efficiency of air filters using acoustics pre-conditioning. Agglomeration is observed in two distinct regions of the test particles (around 0.45 μm and 2 μm) to form agglomerates of larger sizes (0.7 μm and larger than 5 μm) under the acoustics treatment of 140 dB. Specifically, for particles in the range of 0.4 to 0.5 μm in diameter, the concentration is reduced by almost 16%. The agglomeration process improves the PM_2.5_ filtration of a MERV 13 filter from 88% to 92%. For a MERV 11 filter, filtration efficiency is elevated from 73% to 83%, bringing it closer to the higher efficiency counterpart without the additional pressure drop. In addition, the fan power savings from downgrading of the filters has the potential of offsetting the additional power requirements from the acoustics system. The feasibility study provided directions for further scope of applications using acoustic agglomeration and the successful adoption in ACMV systems would require additional breakthroughs in enhancing the efficiency of particle coagulation. This could be achieved by the lengthening of residence times in the acoustic zone or through the deliberate introduction of turbulence within air ducts. The introduction of benign seed particles or humidity can also be used to enhance particle capture rates and thus the agglomeration process.

## Supporting information

S1 Dataset Particle number concentration and filtration efficiency of filters due to acoustics agglomeration. (XLSX)
